# Systemic IFN-I Synergizes with Topical TLR7/8 Agonists to Suppress Metastatic Tumors

**DOI:** 10.34133/research.0739

**Published:** 2025-06-21

**Authors:** Haiting Xu, Chenghui Wang, Bo Xiao

**Affiliations:** ^1^ State Key Laboratory of Resource Insects, College of Sericulture, Textile, and Biomass Sciences, Southwest University, Chongqing 400715, China.; ^2^ Department of Laboratory Medicine, Key Laboratory for Human Disease Gene Study of Sichuan Province and the Department of Laboratory Medicine, Sichuan Provincial People’s Hospital, University of Electronic Science and Technology of China, Chengdu, Sichuan 611731, China.; ^3^ Department of Pharmacy, Personalized Drug Therapy Key Laboratory of Sichuan Province, Sichuan Academy of Medical Sciences & Sichuan Provincial People’s Hospital, School of Medicine, University of Electronic Science and Technology of China, Chengdu, Sichuan 610054, China.

## Abstract

Advancing targeted cancer immunotherapy is pivotal for overcoming distant metastasis and tumor relapse. The recent study in *Nature Cancer* by Sanlorenzo et al. demonstrates a breakthrough strategy combining systemic type I interferon with topical Toll-like receptor 7/8 agonists, where oral imiquimod primes plasmacytoid dendritic cells (DCs) to produce type I interferon, thereby sensitizing conventional DCs in tumors to local Toll-like receptor 7 activation. This approach triggers c-Jun-dependent IL-12 production and CCL2-mediated plasmacytoid DC recruitment, enabling localized and systemic tumor suppression. Importantly, the therapy synergizes with PD-1 blockade to prevent recurrence, representing a significant advance in DC-targeted cancer immunotherapy.

Toll-like receptor 7/8 (TLR7/8) agonists, such as imiquimod (IMQ), with topical applications (e.g., 5% cream) have already been used in the clinic for treating various localized cancers (e.g., basal cell carcinoma). However, due to limited skin penetration, IMQ cream is suitable only for superficial skin tumors [1]. In cases of high-grade malignancies, including malignant melanoma, even if superficially located, topical treatment alone is often inadequate. Alternatively, systemic IMQ administration broadly activates TLR7/8, triggering a cytokine storm characterized by a rapid surge in pro-inflammatory cytokines. Moreover, its short half-life necessitates frequent dosing, which exacerbates toxicity [2]. Dendritic cells (DCs) play a pivotal role in cancer immunotherapy. The activation of DCs by IMQ induces robust immune responses that are capable of suppressing the growth of local tumors [3]. Nonetheless, the specific cellular mechanisms underlying the antitumor immunity mediated by IMQ in DCs are still not fully understood.

Recently, a study by Sanlorenzo et al.’s group [4], published in *Nature Cancer*, demonstrated a novel therapeutic strategy and mechanism, the simultaneous oral and topical administration of IMQ, for treating topically accessible tumors through c-Jun-mediated IL-12 induction in classic DCs (cDCs) activated by type I interferon (IFN-I) from plasmacytoid DCs (pDCs). This strategy offers a promising approach for overcoming the treatment challenges of distant metastasis and tumor relapse (Figure A). cDCs and pDCs are known to be functionally distinct. cDCs are primarily responsible for antigen presentation and T-cell priming [5], while pDCs are specialized in producing IFN-I in response to pathogens [6]. Based on these, the authors innovatively revealed that pDCs were essential for systemic IFN-I production when responding to oral IMQ, and the elevated IFN-I was critical for antitumor outcomes, as demonstrated by the loss of tumor control in pDC-depleted mice (Figure B) and those lacking the IFN-I receptor (Figure C). They further indicated that systemic administration of IFN-I could substitute for oral IMQ, restoring the antitumor effect in pDC-depleted mice when combined with topical IMQ (Figure D). This study demonstrates that systemic administration of IFN-I may replace oral IMQ to elicit effective antitumor responses.

**Figure.**
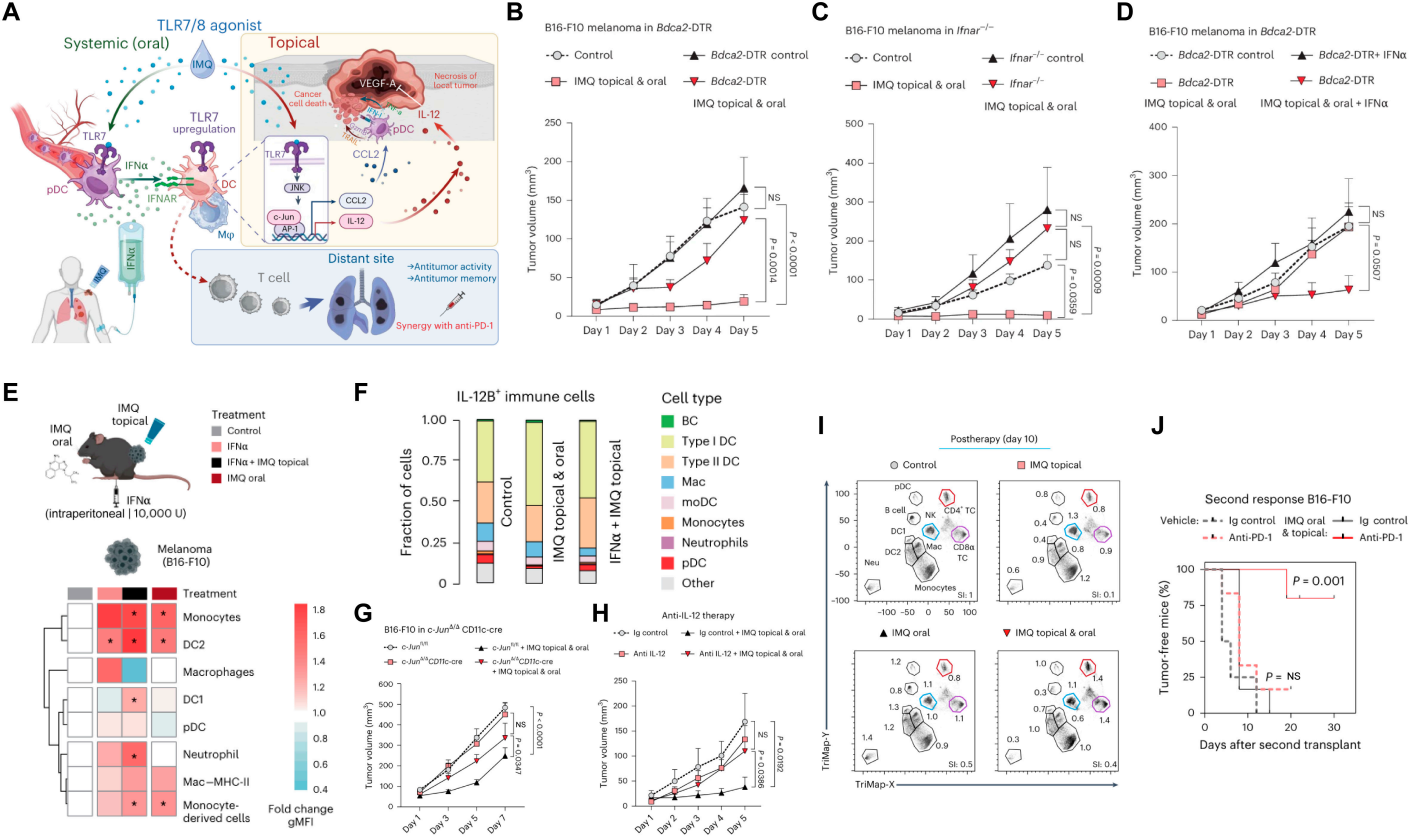
Combined systemic and topical imiquimod (IMQ) enhances both local and distant antitumor responses. (A) Graphical abstract: topical and systemic IMQ activates dendritic cells (DCs) in the tumor microenvironment (TME) to produce IL-12 and CCL2, enhancing CD8^+^ T-cell-driven antitumor responses and synergizing with PD-1 blockades. (B) Tumor progression dynamics (B16-F10) were evaluated in plasmacytoid DC (pDC)-depleted mice (*Bdca2*-DTR) receiving combined topical and oral IMQ. pDCs were specifically depleted in the *Bdca2*-DTR mouse model by using the *Bdca2* promoter to regulate diphtheria toxin receptor (DTR) expression, followed by diphtheria toxin injection. (C) Tumor growth (B16-F10) was monitored in IFN-I-receptor-deficient mice (*Ifnar*^−/−^). (D) Tumor growth profiles (B16-F10) were monitored in pDC-depleted mice (*Bdca2*-DTR). (E) Mice received IFNα and/or topical IMQ or oral IMQ before the analysis of Toll-like receptor 7 (TLR7) expression on myeloid cells by intracellular flow cytometry. The heatmap depicts TLR7 expression on myeloid cells within B16-F10 tumors. gMFI, geometric mean fluorescence intensity. (F) Cells positive for IL-12B fractions of B16-F10 tumors after different treatments. (G) Tumor growth profiles were monitored in *c*-*Jun*^fl/fl^ and *c*-*Jun*^Δ/Δ^ CD11c-Cre mice, revealing that Cre recombinase was expressed specifically in DCs with high CD11c expression, leading to targeted deletion of the *c*-*Jun* gene in these cells. (H) Tumor progression in wild-type mice with B16-F10 melanoma implants was monitored after receiving topical and oral IMQ treatment, with or without anti-IL-12 antibody. (I) TriMap plot of immune cells (CD45^+^) in B16-F10 tumor-bearing mice posttherapy (day 10). SI, similarity index. (J) Survival curves showing tumor-free survival post-B16-F10 rechallenge, with mice given anti-PD-1 antibodies every other day (3 doses) and/or 5 d of combination therapy prior to resection. Reprinted with permission from Ref. [4]. Copyright © 2025, Author(s) Limited. NS, not significant.

Given previous studies showing that adaptive immune cells, such as T and B cells, are dispensable for the antitumor effects during the first week of IMQ treatment [7], the authors focused on myeloid cells, which play a central role in early antitumor immunity. Strikingly, they found that the combination of systemic IFNα and local IMQ administration markedly upregulated TLR7 expression on cDCs (cDC1 and cDC2) within the tumor microenvironment (TME) (Figure E). This further verified that the synergy between oral and local IMQ was based on oral-IMQ-induced IFN-I production by pDCs, which in turn led to the upregulation of TLR7 on cDCs in the TME, thereby sensitizing these immune cells to local IMQ treatment at the tumor site. Subsequently, the authors performed single-cell RNA sequencing on locally treated (accessible) tumor sites to uncover the molecular pathways and effector cells responsible for tumor suppression. The results revealed that both cDC1 and cDC2 were the primary producers of IL-12B in response to the combination therapy (Figure F). IL-12 is well-known for its potency to inhibit tumor-associated angiogenesis [8]. Further genetic ablation experiments demonstrated that *c-Jun* in cDCs functioned downstream of TLR7 signaling to mediate the antitumor effect of IL-12 while simultaneously inducing the secretion of CCL2, which recruited pDCs to the TME for direct tumor suppression (Figure E and H). Although this combinatorial approach did not rely on T or B cells for primary tumor control, it promoted CD8^+^ T-cell responses at distant tumor sites during the later treatment stages (Figure I). Moreover, in 80% of the B16-F10 melanoma model mice, incorporating anti-PD-1 antibodies led to complete prevention of tumor recurrence (Figure J), demonstrating a synergistic interaction between innate immune activation and checkpoint inhibition.

This exciting work employed a combined strategy of oral/systemic IFN-I and topical IMQ application. This approach effectively eliminates primary tumors while also inducing robust immune memory to prevent metastasis and tumor recurrence. Mechanistically, the authors reveal that this combination therapy relies on IFN-I production by pDCs. Further, IFN-I exerts a profound effect on the TME by upregulating TLR7 expression on cDCs in both mice and patients. These sensitized cDCs respond to topical IMQ via the TLR7–c-Jun signaling axis to produce IL-12. The generated IL-12 blocks tumor angiogenesis and induces local tumor necrosis and CCL2, which recruits pDCs to the tumor site for exerting direct cytotoxic activity against tumor cells. These findings lay the foundation for future research on the role of TLR7/8 agonists in maintaining local and systemic immune homeostasis. It is worth noting that this study lacks a comprehensive validation system in the mechanistic investigations. For example, the authors revealed that IFNα could increase the messenger RNA levels of *Tlr7* and *Tlr8* in bone-marrow-derived DCs and macrophages. However, they only used *Tlr7*^−/−^ bone-marrow-derived DCs to demonstrate impaired IL-12B induction. This incomplete genetic validation leaves it unclear whether the observed effects are mediated by TLR7, TLR8, or both. Additionally, the pDC depletion experiments rely on *Bdca2*-DTR mice, a model known to potentially cause off-target effects. Moreover, the study does not include verification using pDC-specific markers (e.g., Siglec-H) to confirm the efficiency and specificity of pDC depletion.

Considering the systemic adverse effects associated with IMQ, the authors demonstrated that systemic IFN-I could functionally substitute for pDCs. When combined with topical IMQ, this collaborative approach achieved complete antitumor responses. However, the clinical application of systemic IFN-I remains limited due to its toxicities, including bone marrow suppression and neuropsychiatric side effect [9]. Although they proposed a short-term IFN-I treatment regimen to mitigate these adverse events, no systematic screening and optimization of dosage and timing were conducted to validate this strategy.

Notably, micro- and nano-delivery systems (e.g., liposomes, micelles, and exosomes) hold great promise in overcoming the key limitations associated with systemic administration of IMQ and IFN-I [10]. These systems can be surface-functionalized with ligands, such as antibodies, peptides, and glycans, to achieve active targeting to the lymph nodes, TME, and DCs. This targeted strategy minimizes off-target distribution to nonimmune organs, reducing nonspecific inflammatory responses and systemic toxicity [11,12]. Controlled-release design, such as pH-sensitive micelles, can prolong the half-life of IMQ and prevent cytokine storms caused by burst drug release [13]. Moreover, micro- and nano-delivery platforms enable the codelivery of IMQ or IFN-I with other therapeutic agents, including tumor antigens, immune checkpoint inhibitors, and chemotherapeutics, promoting synergistic immune activation while reducing the required dosages [14]. These strategies not only mitigate systemic inflammation and related adverse effects but also convert free immunostimulatory agents into more efficient immune activators, which may represent a promising research direction. It is worth mentioning that the translation from laboratory research to clinical application remains one of the major challenges in the field of micro- and nano-delivery systems. First, selecting clinically validated materials (e.g., Food and Drug Administration-approved lipids and polymers) and employing scalable synthesis techniques (e.g., microfluidics and high-pressure homogenization) can facilitate the Investigational New Drug application process [15]. Second, establishing standardized physicochemical parameters—such as particle size, size distribution, drug loading efficiency, release kinetics, and surface charge—along with reliable analytical methods (e.g., dynamic light scattering, high-performance liquid chromatography, and transmission electron microscope) is critical for good manufacturing practice production and quality control [16]. In addition, drug stability and storage conditions have to be carefully addressed [17].
